# 

**Published:** 1872-08

**Authors:** 


☞ To those of our readers (if any such there be) who may be looking so low as to desire to find in a medical journal merely a collection of prescriptions or recipes, that are said to be "good" for certain diseases, we have to say, that we have a higher aim than this; and instead of mere statements (in many cases, very erroneous, if not from intent, from delusion or ignorance) in regard to the application of remedies, we propose to endeavor to keep pace with medicine as a science, and constantly to present expositions of the principles involved in the pathology and therapy of diseases—referring those who have no higher aspirations than to become mere routine practitioners, to "Dr. Gunn's Domestic Medicine," "A Treatise on the Virtues of Vinegar Bitters," *et id omne genus!*

---

**The Editorial and Proprietary Departments of this Journal are entirely *distinct*. Communications connected with the Editorial Department will be directed to the Editors, and those on business will be directed to the Atlanta Medical and Surgical Journal, care of "Plantation Publishing Company."**

☞ *The Editors are not responsible for the views of Contributors.*

